# Generation of the salicylic acid deficient *Arabidopsis* via a synthetic salicylic acid hydroxylase expression cassette

**DOI:** 10.1186/s13007-022-00922-x

**Published:** 2022-06-28

**Authors:** Zilin Cai, Hao Guo, Shijing Shen, Qilu Yu, Jinbin Wang, Engao Zhu, Pinghua Zhang, Lili Song, Yanjun Zhang, Kewei Zhang

**Affiliations:** 1 Zhejiang Provincial Key Laboratory of Biotechnology on Specialty Economic Plants, College of Chemistry and Life Sciences, Institute of Plant Stress Adaptation and Genetic Enhancement, Zhejiang Normal University, Jinhua, 321004 Zhejiang People’s Republic of China; 2 State Key Laboratory of Subtropical Silviculture, Sino-Australia Plant Cell Wall Research Centre, School of Forestry and Biotechnology, Zhejiang A&F University, Lin’an, Hangzhou, 311300 Zhejiang People’s Republic of China

**Keywords:** Salicylic acid, Salicylic acid hydroxylase, Synthetic gene expression cassette, NahG, Pathogen resistance, Leaf senescence

## Abstract

**Background:**

Salicylic acid (SA) is one of the plant hormones, which plays crucial roles in signaling transduction in plant growth, disease resistance, and leaf senescence. *Arabidopsis* (*Arabidopsis thaliana*) SA 3-hydroxylase (S3H) and 5-hydroxylase (S5H) are key enzymes which maintain SA homeostasis by catalyzing SA to 2,3-dihydroxybenzoic acid (DHBA) and 2,5-DHBA, respectively.

**Results:**

SA deficient transgenic *Arabidopsis* lines were generated by introducing two binary vectors *S5Hpro::EGFP-S3H* and *35Spro::EGFP-S3H* respectively, in which the expression of *S3H* is under the control of the *S5H* promoter or CaMV 35S promoter. Compared with the constitutive expression of *S3H* gene under the control of 35S promoter, the *S3H* gene under the native *S5H* promoter is activated by endogenous SA and results in a dynamic control of SA catabolism in a feedback mode. The SA accumulation, growth, leaf senescence, and pathogen resistance of the *S5Hpro::GFP-S3H* transgenic plants were investigated in parallel with *NahG* transgenic plants. The SA levels in the *S5Hpro::EGFP-S3H* transgenic plants were similar to or slightly lower than those of *NahG* transgenic *Arabidopsis* and resulted in SA deficient phenotypes. The low-SA trait of the *S5Hpro::EGFP-S3H* transgenic lines was inherited stably in the later generations.

**Conclusions:**

Compared with *NahG* transgenic lines producing by-product catechol, *S5Hpro::EGFP-S3H* transgenic lines reduce SA levels by converting SA to a native product 2,3-DHBA for catabolism. Together, we provide new SA-deficient germplasms for the investigations of SA signaling in plant development, leaf senescence, and disease resistance.

**Supplementary Information:**

The online version contains supplementary material available at 10.1186/s13007-022-00922-x.

## Background

Salicylic acid (SA) is an important hormone in plants. The biosynthesis of SA is generally increased after pathogen infection and is tightly related to plant immunity [1, 2]. It upregulates the expression of *pathogenesis-related* (*PR*) genes through the NONEXPRESSER OF PR GENES (NPR) proteins mediated signal pathway [3]. In addition, SA is also involved in plant growth and development processes, including seed germination [4, 5], flowering [6–8], and leaf senescence [9–11]. Previous studies have shown that SA can regulate plant growth and development by cross-talking with other plant hormones. For example, *Arabidopsis *(*Arabidopsis thaliana*) NPR1 was found to interact with the core transcription factor of ethylene signaling pathway, ETHYLENE INSENSITIVE 3 (EIN3), and inhibit the formation of apical hooks [12] and promote leaf senescence [13, 14]. Moreover, SA targets to the A subunit of protein phosphatase 2A (PP2A) and inhibits the activity of PP2A, thereby inhibits auxin transport and root development, including growth, gravitropic response, and lateral root organogenesis, through changing the polarity distribution of PIN-FORMED 2 (PIN2) [15].

*Arabidopsis* produces SA through the isochorismate synthase (ICS) and the phenylalanine ammonia-lyase (PAL) pathways [16]. In *Arabidopsis*, chorismate can be converted into SA via ICS pathway in a two- or three- step processes involving ICS1, PBS3, or EPS1 [1, 17, 18]. In rice, phenylalanine is converted to *trans*-cinnamic acid by PALs, and serves as a precursor for a 3-hydroxyacyl-CoA dehydrogenase [ABNORMAL INFLORESCENCE MERISTEM1 (AIM1)] to synthesis benzoic acid (BA) [19] and then are converted to SA by an uncharacterized enzyme [20]. After synthesis, SA is modified into different SA derivatives in plants, such as hydroxylation, glycosylation, methylation, and amino acid conjugation, which may serve as the transportation or storage forms and affect the SA homeostasis [2]. Among them, SA hydroxylation was considered to be one of the major pathways for SA catabolism [21, 22].

In *Arabidopsis*, there are two forms of SA hydroxylation, 2,3-dihydroxybenzoic acid (DHBA) and 2,5-DHBA, which are hydroxylated on the 3rd and 5th C atoms of benzoic acid by SA 3-hydroxylase (S3H) and SA 5-hydroxylase [S5H, is also called downy mildew resistant 6 (DMR6)], respectively [21]. S3H catalyzes SA to 2,3-DHBA and *S3H* gene is specially expressed in mature or senescing plants [22]. The lack of *Arabidopsis S3H* increases SA level in the senescence stage and results in early leaf senescence phenotype [22]. This indicates that S3H participates in the SA catabolism pathway during *Arabidopsis* leaf senescence. Different from S3H, S5H/DMR6 catalyzes SA to 2,5-DHBA and the gene is continuously expressed throughout the life cycle of plants and is induced by pathogens and leaf senescence [23]. The *s5h/dmr6* mutant and its double mutant with *s3h* accumulated high levels of SA and resulted in a constitutive defense response and a dwarfed morphology [23, 24], indicating that S5H/DMR6 affects the trade-off between growth and immunity [23]. Although both *S3H* and *S5H/DMR6* were induced by SA or pathogens, the *S5H/DMR6* expression is more sensitive (> 10 times) to SA and pathogen treatments than *S3H* [22, 23]. Interestingly, at biochemical level, S5H/DMR6 has substrate inhibitory properties, while S3H does not exhibit substrate inhibitory properties [23].

NahG is a salicylate hydroxylase purified from *Pseudomonas putida*. Different from S3H and S5H hydroxylase of *Arabidopsis*, it catalyzes the hydroxylation of SA to produce catechol [25]. Overexpression of *NahG* gene in tobacco (*Nicotiana tabacum*) or *Arabidopsis* resulted in significantly decrease in SA level, loss of systemic acquired resistance (SAR), and increased susceptibility to viruses, fungi and pathogens [26–28]. Due to the defect of disease resistance, *NahG* transgenic plants have been widely used in the studies on disease resistance [29, 30]. Since SA induces senescence-associated gene (SAG) expression in leaf senescence [31], *NahG* transgenic plants have been used to study the SA signaling pathways in leaf senescence [9, 11]. In addition, *NahG* transgenic plants have also been used to study flowering [8, 32], stomatal immunity [33], salt tolerance [34, 35], and the cross talk between SA and other plant hormones [5, 36]. Recently, *NahG* transgenic *Arabidopsis* plants have also been used to achieve higher efficiency of *Agrobacterium*-mediated transient transformation for efficient assessing protein subcellular localization or protein–protein interactions [37]. Although *NahG* transgenic plants have been widely used so far, the by-product catechol produced by NahG was suspected to cause side effects [38, 39].

In this study, we designed a synthetic expression cassette *S5Hpro::EGFP-S3H*, in which the native SA hydroxylase gene *S3H* is driven by the native promoter of *S5H* gene. Meanwhile, for comparison, we constructed a vector harboring *35Spro::EGFP-S3H* in which the *S3H* gene is driven by 35S promoter*.* The SA levels in the *S5Hpro::EGFP-S3H* as well as *35Spro::EGFP-S3H* transgenic *Arabidopsis* were both significantly reduced. The disease resistance, growth, and leaf senescence phenotypes of the *S5Hpro::EGFP-S3H* transgenic plants were investigated in parallel with the *NahG* transgenic plants. Collectively, by utilizing a plant-derived SA hydroxylase, we have generated low-SA transgenic lines that can be used to study plant growth and development, stress, and disease resistance.

## Results

### Design of a new strategy to reduce the endogenous SA by overexpressing *Arabidopsis* SA hydroxylase

**Fig. 1:**
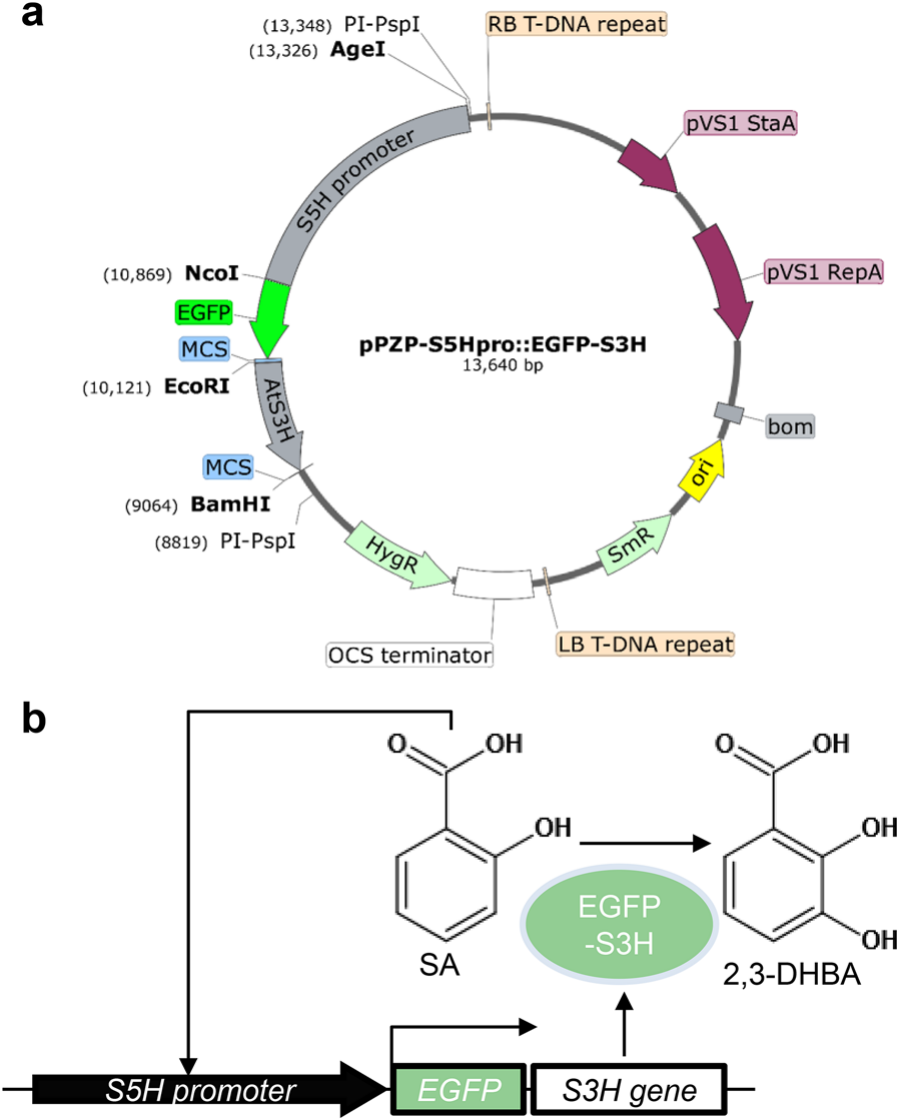
Design of a synthetic SA hydroxylase expression cassette catalyzing SA to 2,3-DHBA. **a** Vector map for *S3H* gene overexpression under *S5H* promoter. The *S5H* promoter of *Arabidopsis* was cloned and constructed into the plant expression vector (pPZP-RCS2) to drive the expression of *EGFP-S3H*. The map was prepared by SnapGene. **b** The principle of a feedback loop for SA catabolism to 2,3-DHBA in the *S5Hpro::EGFP-S3H* transgenic plants. The *S5H* promoter can be induced by SA and then drive the expression of *EGFP-S3H.* The expressed S3H enzyme can convert SA into 2,3-DHBA and reduce the SA levels

To reduce the SA level in *Arabidopsis*, we took advantage of an SA hydroxylase S3H from *Arabidopsis* to construct a synthetic gene cassette. The binary vector pPZP-*S5Hpro::EGFP-S3H* (Fig. 1a) containing the synthetic SA hydroxylase expression cassette was constructed to express S3H enzyme under the promoter of *S5H* gene. For comparison, the binary vector pMDC43-*35Spro::EGFP-S3H* (Additional file 1: Fig. S1a) was constructed to express *S3H* gene under the 35S promoter. Then the vectors were transformed into Col-0 (used as WT) and generated *S5Hpro::EGFP-S3H* and *35Spro::EGFP-S3H* transgenic plants, respectively. In the *S5Hpro::EGFP-S3H* transgenic plants, the *S5H* promoter can be induced by endogenous SA, then drive the expression of *EGFP-S3H*, which can convert SA to 2,3-DHBA for repressing the SA accumulation. Compared with the constitutive expression of *EGFP-S3H* under 35S promoter (Additional file 1: Fig. S1b), this forms a strong feedback loop to maintain SA homeostasis at low levels in *Arabidopsis* (Fig. 1b).

### SA level is significantly reduced in the *S5Hpro::EGFP-S3H* transgenic plants

Similar to *NahG* transgenic plants, the rosette leaf diameters of *S5Hpro::EGFP-S3H* and *35Spro::EGFP-S3H* transgenic plants were both larger than WT (Additional file 1: Fig. S2a, b). The levels of total SA in the *S5Hpro::EGFP-S3H*, *35Spro::EGFP-S3H*, and the *NahG* transgenic plants were all significantly lower than that of WT (Additional file 1: Fig. S2c–f). The average SA levels in 4 independent lines of *35Spro::EGFP-S3H* were reduced to 23.2% of WT and the average SA levels in 7 independent lines of *S5Hpro::EGFP-S3H* were reduced to 11.9% of WT, suggesting the SA reduction in the *S5Hpro::EGFP-S3H* transgenic plants seems lower than that in *35Spro::EGFP-S3H* transgenic plants (Additional file 1: Fig. S2c–f). Thereby the *EGFP-S3H* expression under *S5H* promoter is suitable for generating SA deficient *Arabidopsis.*

**Fig. 2:**
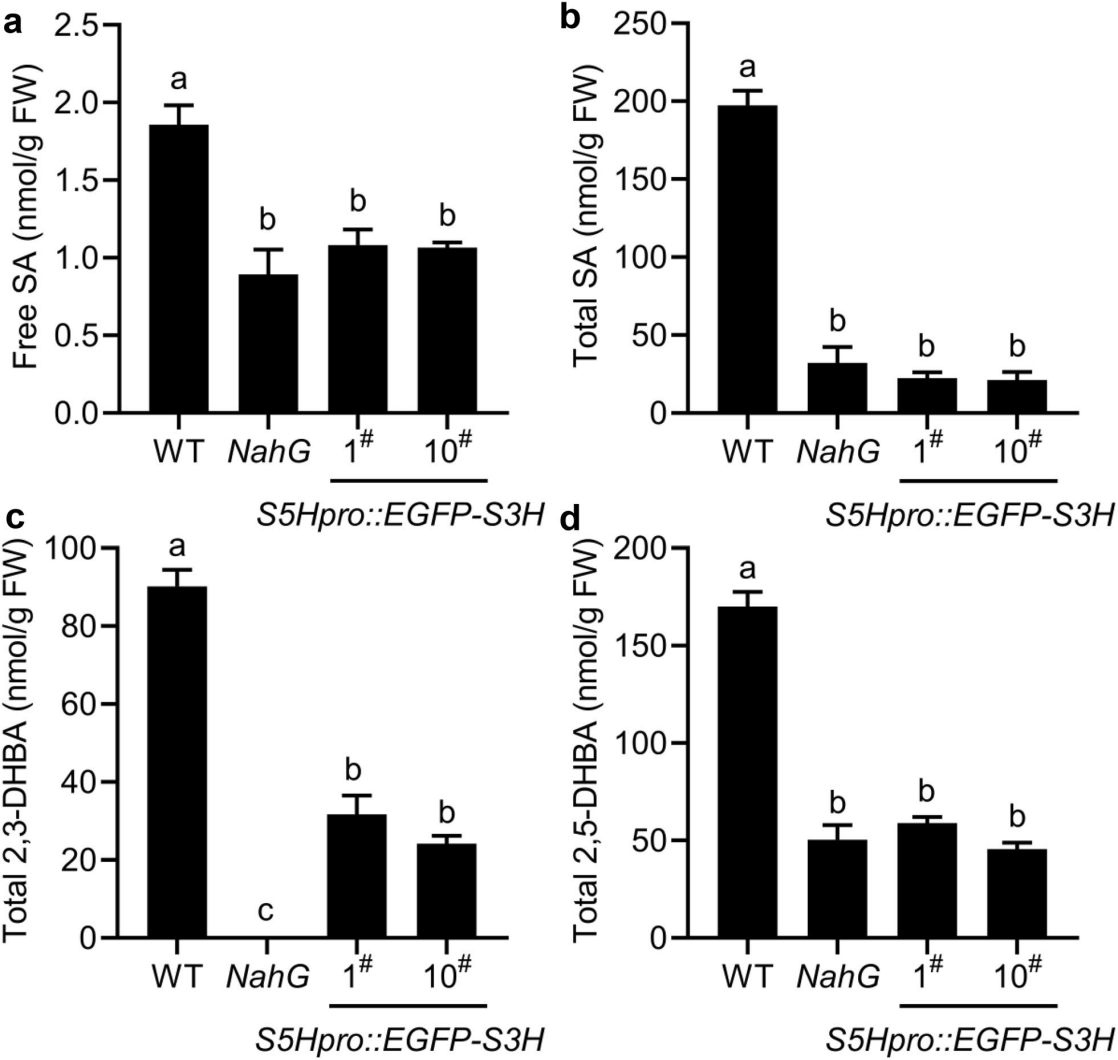
Quantification of SA, 2,3-DHBA and 2,5-DHBA in the *S5Hpro::EGFP*-*S3H* transgenic plants. The levels of free SA (**a**), total SA (**b**), total 2,3-DHBA (**c**), and total 2,5-DHBA (**d**) in WT, *NahG* and the single-copy *S5Hpro::EGFP-S3H* transgenic plants at 28 days after germination (DAG). The data are means ± SE (n = 3 biological replications); *FW* fresh weight. Statistical differences among replicates are labeled with different letters (*P* < 0.05, one-way ANOVA and post-hoc Tukey’s test)

To generate a stable plant with low abundance of SA for genetic studies, we selected the single-copy insertion lines based on the hygromycin B resistance in the MS medium as described in the methods [40, 41]. We checked the *S3H* gene expression in the transgenic plants by qRT-PCR and found the *S3H* expression in the transgenic plants is slightly higher than that in the WT (Additional file 1: Fig. S3). The levels of SA, 2,3-DHBA and 2,5-DHBA in the *S5Hpro::EGFP-S3H* transgenic plants were quantified by HPLC. The results indicated that the free SA and total SA levels in *S5Hpro::EGFP-S3H* were reduced to  ~ 58% and  ~ 11% of those in WT, respectively, which displayed similar pattern to *NahG* transgenic *Arabidopsis* (Fig. 2a, b). The total 2,3-DHBA levels were reduced to  ~ 27% of WT level in the *S5Hpro::EGFP-S3H* transgenic plants and were not detected in *NahG* transgenic plants (Fig. 2c). In addition, the total 2,5-DHBA levels in the *S5Hpro::EGFP-S3H* transgenic plants were reduced to ~ 35% of WT level, which is similar to that in *NahG* transgenic plants (Fig. 2d). Together, overexpression of a single-copy *S3H* under the control of *S5H* promoter significantly reduced the levels of SA and SA hydroxylated products 2,3-DHBA/2,5-DHBA. The single-copy *S5Hpro::EGFP-S3H* transgenic plants were used for the further studies of the low-SA traits such as growth, leaf senescence, and pathogen resistance.

### Growth of the *S5Hpro::EGFP-S3H* transgenic plants

**Fig. 3:**
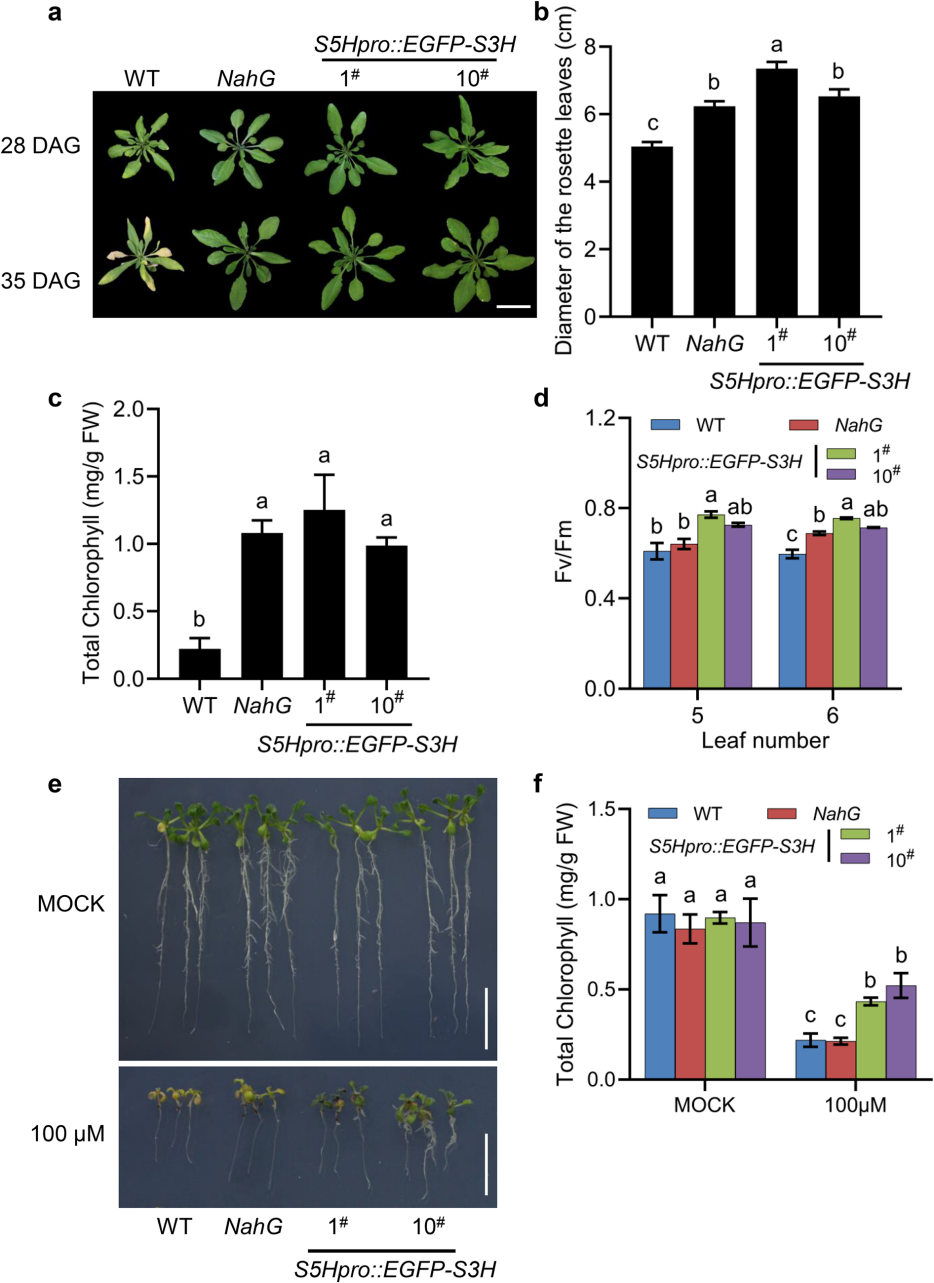
Growth and leaf senescence phenotypes of the *S5Hpro::EGFP-S3H* transgenic plants. **a** Morphological phenotypes of WT, *NahG* and *S5Hpro::EGFP-S3H* transgenic plants at 28 and 35 DAG, Bar = 2 cm. **b** Quantification of the *S3H* expression in WT, *NahG* and *S5Hpro::EGFP-S3H* transgenic plants at 21 DAG by qPCR. The data are means ± SE (n = 3 biological replications). **c** Quantification of the rosette leaf diameters from plant of (**a**) at 28 DAG. The data are presented as means ± SE (n ≥ 10 biological replications). **d** Quantification of chlorophyll content in the 5–6th leaves from plant of (**a**) at 35 DAG, the data are means ± SE (n = 4 biological replicates); *FW*, fresh weight. **e** Fv/Fm of the 5–6th leaves from plant (**a**) at 35 DAG, the data are means ± SE (n = 4 biological replicates). **e** Phenotypes of WT, *NahG* and *S5Hpro::EGFP-S3H* transgenic plants grown on 1/2 MS medium with or without 100 μM sodium salicylate. Bar = 1 cm. **f** Quantification of chlorophyll content from plant of (**e**), the data are means ± SE (n = 4 biological replications); *FW*, fresh weight. Statistical differences among replicates are labeled with different letters (*P* < 0.05, one-way ANOVA and post-hoc Tukey’s test)

We further investigated the growth of the low-SA transgenic plants with the *NahG* transgenic plants as a control. Compared with WT, the rosette leaf size of the *S5Hpro::EGFP-S3H* transgenic plants was significantly increased. At 28 days after germination (DAG), the average rosette leaf diameter of *S5Hpro::EGFP-S3H* transgenic plants was  ~ 37.5% higher than that of WT (Fig. 3a, b). Furthermore, the pPZP-*S5Hpro::EGFP-S3H* vector was also successfully transformed into Wassilewskija (Ws) and Landsberg *erecta* (L*er*) accession *Arabidopsis* respectively. Similar to *S5Hpro::EGFP-S3H* transgenic plants, the rosette leaf size of the two transgenic plants was also significantly increased (Additional file 1: Fig. S4a, b). At 28 DAG, the average rosette leaf diameter of S5Hpro::EGFP-S3H/Ws transgenic plants was  ~ 47.5% larger than that of WT, while that of S5Hpro::EGFP-S3H/L*er* transgenic plants was  ~ 41.4% larger (Additional file 1: Fig. S4c, d). The results suggested that the growth of the rosette leaves of *S5Hpro::EGFP-S3H* transgenic plants in difference accession backgrounds was all strongly promoted due to SA deficiency.

### Leaf senescence and the ability to detoxify SA of the *S5Hpro::EGFP-S3H* transgenic plants

SA is an important plant hormone involved in *Arabidopsis* leaf senescence [9, 42]. To further investigate whether *S3H* overexpression affects the normal leaf senescence process, we observed the phenotypes of *S5Hpro::EGFP-S3H* transgenic plants at the senescence stage. The results showed that the rosette leaf margins of WT turned yellowed at 35 DAG under our condition; especially the old leaves had a severe senescence phenotype. In contrast, *S5Hpro::EGFP-S3H* and *NahG* transgenic plants exhibited delayed leaf senescence phenotype (Fig. 3a). Chlorophyll content and Fv/Fm value of the rosette leaves were measured at 35 DAG. The results showed that the total chlorophyll contents of the 5th and 6th rosette leaves of the *S5Hpro::EGFP-S3H* transgenic plants and *NahG* transgenic plants were significantly higher than that of WT (Fig. 3c). Consistently, the chlorophyll fluorescence parameter Fv/Fm of the 5th and 6th rosette leaves of *S5Hpro::EGFP-S3H* transgenic plants and *NahG* transgenic plants were also higher than that of WT (Fig. 3d). These results indicated that, similarly to the *NahG* transgenic plant, the *S5Hpro::EGFP-S3H* transgenic *Arabidopsis* plants significantly delay leaf senescence.

To check the plant ability to detoxify SA, the *S5Hpro::EGFP-S3H* transgenic plants were grown in half MS medium with or without 100 μM SA in parallel to WT and *NahG* transgenic plants. After 7-days treatment with SA, the WT and *NahG* transgenic plants were dead while the *S5Hpro::EGFP-S3H* transgenic plants were still alive (Fig. 3e, f). The results showed the *S5Hpro::EGFP-S3H* transgenic plants had stronger ability to detoxify SA than that of *NahG* transgenic plants and WT.

### Pathogen resistance reduction of the *S5Hpro::EGFP-S3H* transgenic plants

**Fig. 4:**
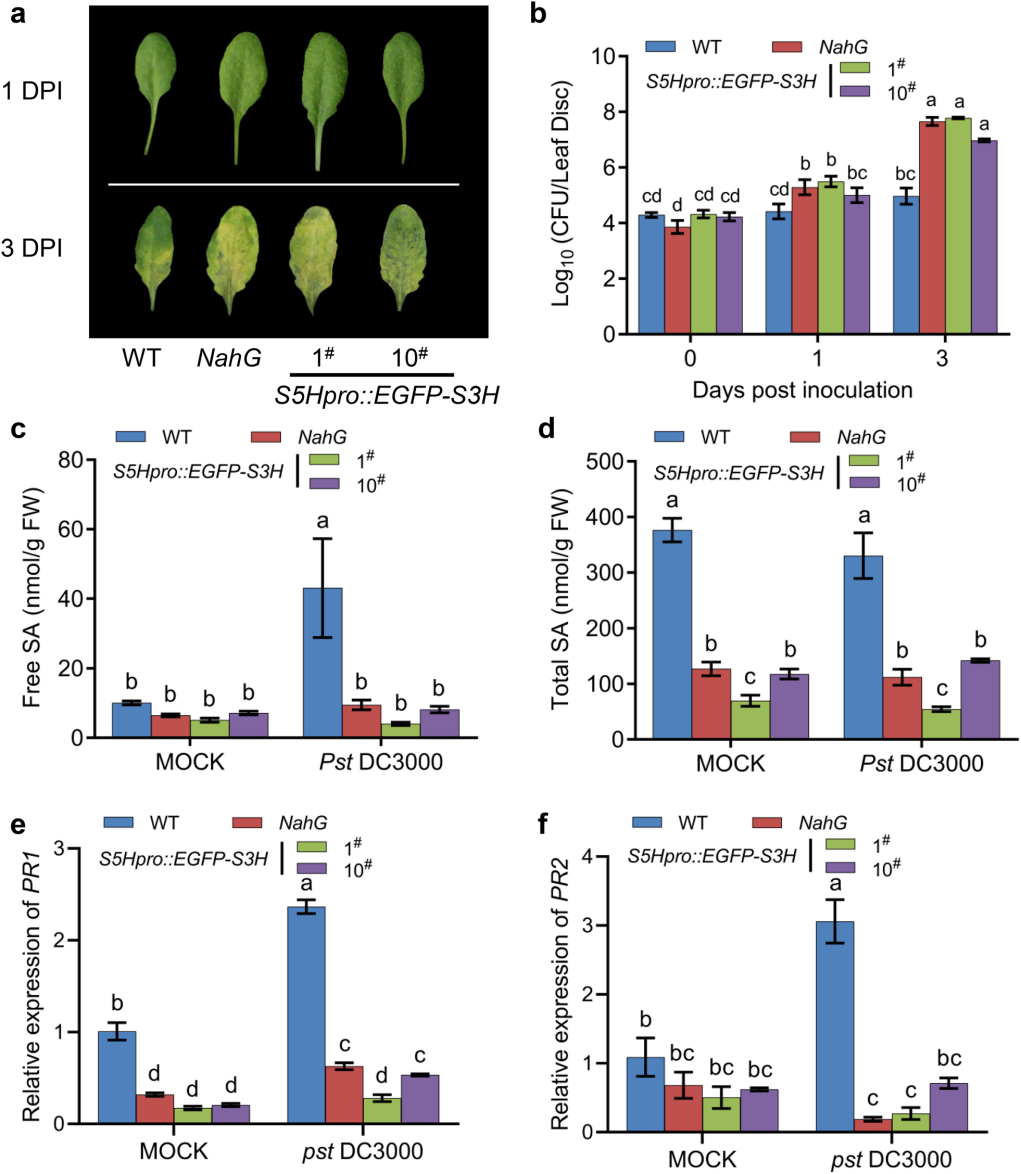
Pathogen resistance of the *S5Hpro::EGFP-S3H* transgenic plants to *Pst* DC3000. **a** The disease symptoms of WT, *NahG*, single-copy *S5Hpro::EGFP-S3H* transgenic plants at 1 and 3 days after *Pst* DC3000 infection. **b** Quantification of the growth of *Pst* DC3000 in plants of **a** at 0, 1, 3 days post inoculation (DPI). The data are means ± SE (n = 6 biological replications). The levels of free SA (**c**) and total SA (**d**) in WT, *NahG* and *S5Hpro::EGFP-S3H* transgenic plants after *Pst* DC3000 infection. The data are means ± SE (n = 4 biological replications). Quantification of the *PR1* (**e**) and *PR2* (**f**) expression in WT, *NahG* and *S5Hpro::EGFP-S3H* transgenic plants after *Pst* DC3000 infection by qPCR. The data are means ± SE (n = 3 biological replications). Statistical differences among replicates are labeled with different letters (*P* < 0.05, one-way ANOVA and post-hoc Tukey’s test)

To explore whether overexpression of *S3H* reduces the disease resistance, we treated WT, *NahG* and *S5Hpro::EGFP-S3H* transgenic plants with *Pseudomonas syringae* pv. *tomato* strain DC3000 (*Pst* DC3000) and checked the bacterium growth at 0, 1 and 3 days. In parallel with *NahG* transgenic plants, *S5Hpro::EGFP-S3H* transgenic plants were more susceptible to *Pst* DC3000 than WT (Fig. 4a, b). The leaves of both the transgenic plants showed disease spots at three days after inoculation (DPI) (Fig. 4a). Consistently, the pathogen growth in the *S5Hpro::EGFP-S3H* and *NahG* transgenic plants was significantly accelerated compared with that in WT at 1 and 3 DPI (Fig. 4b). Meanwhile, the SA levels and *PR* gene (*PR1* and *PR2*) expression were respectively quantified in WT, *S5Hpro::EGFP-S3H* and *NahG* transgenic plants at 24 h after pathogen inoculation. Both the SA accumulation (Fig. 4c, d) and *PR* gene expression (Fig. 4e, f) were significantly suppressed in *S5Hpro::EGFP-S3H* and *NahG* transgenic plants compared with that of WT. These data indicated that the *S5Hpro::EGFP-S3H* transgenic plants are suitable for plant pathology studies.

### Low-SA trait is stably inherited in the *S5Hpro::EGFP-S3H* transgenic plants

**Fig. 5:**
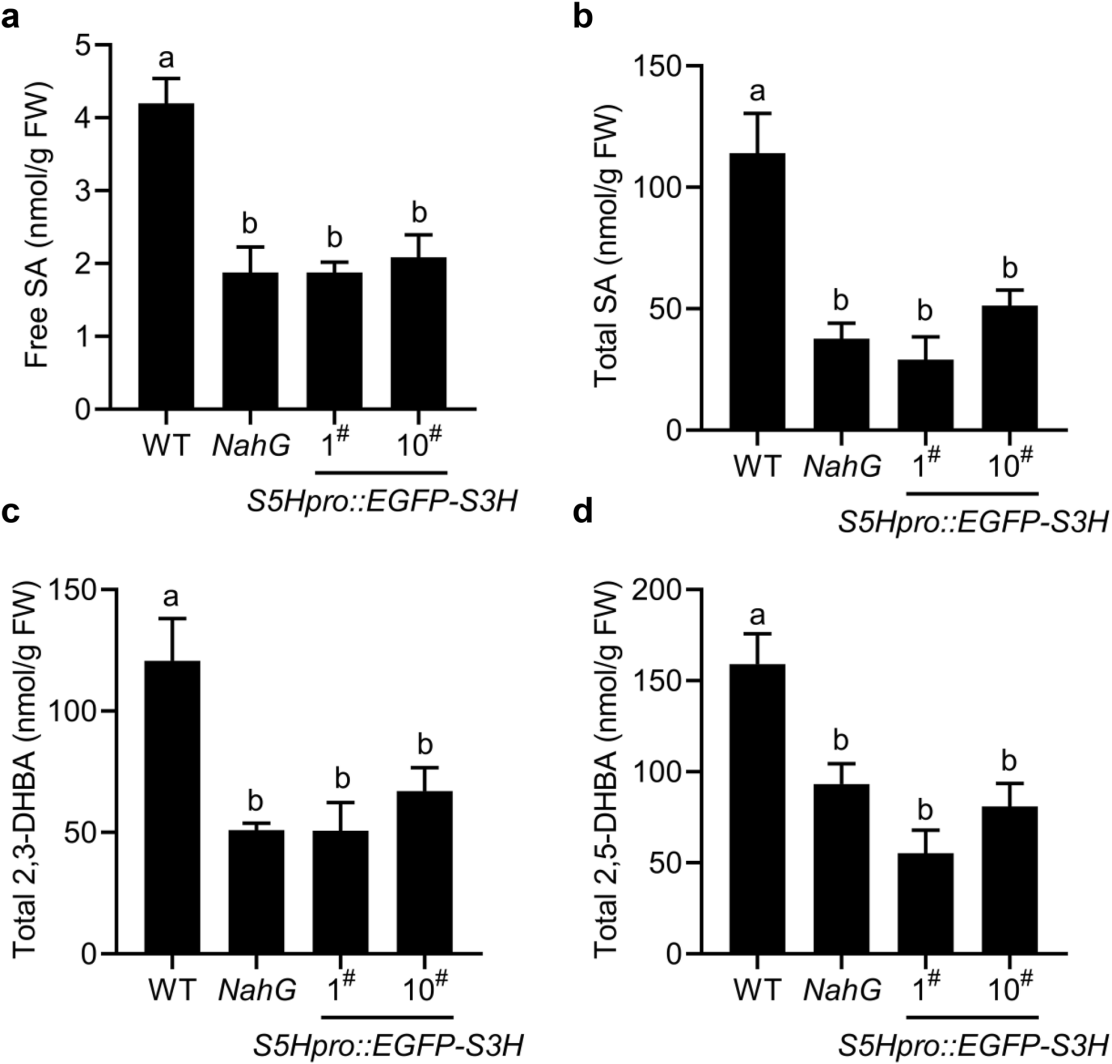
Quantification of SA, 2,3-DHBA and 2,5-DHBA in *S5Hpro::EGFP-S3H* transgenic plants at T5 generation. Quantification of free SA (**a**), total SA (**b**), total 2,3-DHBA (**c**) and total 2,5-DHBA (**d**) from WT, *NahG*, and single-copy *S5Hpro::EGFP-S3H* transgenic lines at T5 generation at 35 DAG. The data are means ± SE (n = 4 biological replications); *FW* fresh weight. Statistical differences among replicates are labeled with different letters (*P* < 0.05, one-way ANOVA and post-hoc Tukey’s test)

A number of factors, such as the number of copies inserted and the influence of the external environment, can alter gene inheritance and expression, and even prevent the expression of exogenous genes [40, 41, 43]. To know whether the low-SA trait of the *S5Hpro::EGFP-S3H* transgenic plants is stably inherited in the offspring of the transgenic plants, we checked the SA levels in the T5 generation of the *S5Hpro::EGFP-S3H* transgenic plants. The results showed that the free SA and total SA levels in T5 generation of the *S5Hpro::EGFP-S3H* transgenic plants were similar to those in *NahG* transgenic plants, and were significantly lower than those in WT (Fig. 5a, b). The levels of 2,3-DHBA and 2,5-DHBA in the *S5Hpro::EGFP-S3H* transgenic plants were also significantly lower than that of WT (Fig. 5c, d). Therefore, the low-SA trait of the *S5Hpro::EGFP-S3H* transgenic plants was inherited stably.

## Discussion

Two native SA hydroxylases named S3H and S5H/DMR6 were characterized in *Arabidopsis* [22–24]. Overexpression of *S3H* and *S5H* under 35S promoter in *Arabidopsis* significantly reduces the SA level and results in stronger pathogen resistance, larger leaf size, and delayed leaf senescence [22, 23, 44]. The *S5H*/*DMR6* gene expression is particularly sensitive to SA and pathogen treatment, whereas the S3H enzyme shows a high SA hydroxylase activity without substrate inhibition [23]. Therefore, we attempted to build a synthetic expression cassette *S5Hpro::EGFP-S3H* by using the SA-inducible promoter of *S5H* and SA hydroxylase enzyme S3H. The total SA levels of the *S5Hpro::EGFP-S3H* transgenic plants were significantly decreased at multiple generations, and were similar to *NahG* transgenic plants (Additional file 1: Fig. S2; Figs. 2, 5). Intriguingly, the total 2,3- and 2,5-DHBA levels produced in the *S5Hpro::EGFP-S3H* transgenic were also lower than that in WT (Figs. 2, 5). In addition, the *S3H* gene expression was only slightly higher than that in WT (Additional file 1: Fig. S3). We speculate this could be due to the fact that the total SA level in *S5Hpro::EGFP-S3H* transgenic plants was 9 times lower than that in WT and resulted in low expression of both *S3H* and *S5H* genes and low amount of hydroxylated products 2,3- and 2,5-DHBA. These results showed that the *S5Hpro::EGFP-S3H* transgenic plants can efficiently maintain low levels of SA, hydroxylated SA, and *S3H/S5H* gene expression.

*S5Hpro::EGFP-S3H* transgenic *Arabidopsis* exhibited promoted leaf growth, enhanced susceptibility, and delayed leaf senescence (Figs. 3, 4), which were consistent with the observations in the transgenic plants of *S3H* or *S5H* under 35S promoter [22, 23]. The *S5H* gene was recently characterized to be a direct target gene of TGA transcription factors which are downstream of SA receptors NPR1 and NPR3/NPR4 [45]. Compared to 35S promoter, the *S5H* promoter is dynamic and the activity is increased under conditions which can induce the SA biosynthesis. Since the 35S promoter sometimes is epigenetically modified and results in gene silencing [46, 47], the gene expression under the native promoter of *S5H* is more stably inherited. In addition, *S5H* gene is expressed from young stage to old stage and from root to shoot, and it is strongly induced by various biotic and abiotic stresses (Additional file 1: Fig. S5) [23], suggesting the feedback loop in *S5Hpro::EGFP-S3H* transgenic plants can efficiently maintain the low-SA levels in different tissues and under different conditions. However, the transcription activation of *Arabidopsis S5H* gene promoter in other species remains to be investigated.

Similar to *NahG* transgenic plants, overexpression of *S3H* under native *S5H* promoter in *Arabidopsis* could increase the susceptibility to *Pst* DC3000 (Fig. 4), providing an optional genetic material for the studies of plant immunity. NahG is a bacterial SA hydroxylase which converts SA to catechol, and the by-product catechol in the transgenic plants might cause side effects to the studies [38, 39]. S3H is a plant-derived SA hydroxylase which converts SA to a native product 2,3-DHBA then is converted to sugar conjugated products by UGT76D1 for inactivation [48]. In comparison with *NahG* transgenic plants, the expression of plant derived S3H enzyme under the control of *S5H* promoter in *Arabidopsis* significantly reduced the SA levels without introducing new metabolites. Previously, it was suspected that the susceptibility of *NahG* transgenic plants to non-host pathogen *Pseudomonas syringae* pv. *phaseolicola* NPS3121 (*Psp*) was caused by the production of catechol [39]. These contradicted pathogen resistance of *NahG* transgenic plants to *Psp* and *Pst* DC3000 can be validated in the *S5Hpro::EGFP-S3H* transgenic plants to know the effect of the by-product catechol on plant immunity.

As a genetic material for the community, it is necessary to ensure that the target trait can be stably inherited. The T5 generation of *S5Hpro::EGFP-S3H* transgenic plants were shown to maintain a low SA level (Fig. 5), suggesting that the SA deficient transgenic plants can be stably inherited. Different promoters such as 35S or maize ubiquitin 1(Ubi-1) [49] can also be used to drive *S3H* gene expression to reduce SA levels in different plant species including monocots and dicots for the studies of SA signaling. On the other hand, the materials can be used to study the functions of PAL-pathway mediated SA biosynthesis in the *Arabidopsis ics1 ics2* double mutant background [50]. In addition, the *S5Hpro::EGFP-S3H* overexpression plants are suitable for studying the growth penalty of disease resistance since the enhanced rosette leaf growth and reduced pathogen resistance in these transgenic plants (Figs. 3, 4). In summary, we provide a new way to generate low-SA germplasm for the studies on plant growth and development, leaf senescence, cell death, and stress responses.

## Materials and methods

### Plant materials and growth conditions

*Arabidopsis thaliana* Columbia-0 (Col-0) accession was used as the wild type (WT) in all experiments, while *NahG* transgenic plants were also used as a control for phenotyping and SA metabolism. Seeds were sown on a plant medium containing Murashige and Skoog (1/2 MS) medium with 3% (w/v) sucrose, 0.3% (w/v) plant agar and corresponding antibiotics. Before being moved into a growth chamber with 16-h-light/8-h-dark photoperiod and 22 ℃, seeds were stratified at 4 ℃ for 3 days. Then the plants with two true leaves were moved into the soil and placed in the *Arabidopsis* culture chamber under 16/8 photoperiod with a constant temperature of 22℃.

### Plasmid construction and transformation

The 2.4-kb *S5H/DMR6* promoter were amplified by *S5H*pro-AgeI (5′-CATGACCGGTTCCCAAACCATGATGGCACC-3′) and *S5H*pro-NcoI (5′-CATGCCATGGCAGAAAATTGAAGAAGAATC-3′) primers, and the fragment was cloned into pSAT6-GFP-C1 to form pSAT6-*S5Hpro*::EGFP-C1. Then the *S3H* coding sequence was amplified from the cDNA of WT by using the pair of primers, *S3H*-EcoRI (5′-CGGAATTCTATGGCAACTTCTGCAATATC-3′) and *S3H*-BamHI (5′-CGGGATCCTTAGGTTGTTGGAGCTTTGA-3′), and was cloned into pSAT6-*S5Hpro*::EGFP-C1 to form pSAT6-*S5Hpro::EGFP-S3H*. Finally, the *S5Hpro::EGFP-S3H* expression cassette was cloned into the binary vector pPZP-RCS2 to form binary vectors pPZP-*S5Hpro::EGFP-S3H*.

The *S3H* gene was amplified by *S3H*-F (5′-GCAGGCTCCGAATTC ATGGCAACTTCTGCAATATC-3′) and *S3H*-R (5′-AAGCTGGGTCGAATTC TTAGGTTGTTGGAGCTTTGA-3′) primers, and the fragment was cloned into pCR8 to form pCR8-*S3H* by Sequence and Ligation Independent Cloning (SLIC) [23]. Finally, the pCR8-*S3H* through Gateway LR Cloning reaction cloned into the destination vector pMDC43 to form expression vector pMDC43-*35Spro::EGFP-S3H*.

The plasmids were transformed into WT by *Agrobacterium*-mediated floral dip transformation to generate *S3H* overexpression transgenic plants. The T2 generation plants with a separation ratio of 3:1 (survival: death) on the Hygromycin B (hyg)-resistant medium were selected, and the seeds of the T3 generation which all survived on the hyg-resistant medium were characterized as a single-copy transgenic plant.

### Pathogen treatment

Following the previously described method [25], the pathogen *Pst* DC3000 was used to carry out the susceptibility experiment on the relevant transgenic plants. The plants were grown in the growth chamber with 12-h-light/12-h-dark photoperiod and 22 ℃, and were sprayed with the pathogen suspension containing 0.03% Silwet L-77 at 28 DAG, then kept moisture for 3 days. The growth of the lesions was observed, and the leaf discs were taken with a puncher for colony counting.

### Quantification of SA and hydroxylated SA by HPLC

All the rosette leaves of WT, *NahG*, and *S5Hpro::EGFP-S3H* transgenic plants were extracted for SA metabolic analysis according the previously described method [23]. The rosette leaves were ground to powder in liquid nitrogen. Approximately 100 mg powder was put into a 2-mL Eppendorf tube with 1 mL extraction buffer (80% MeOH containing 50 μM methyl salicylate as an internal standard). After agitated for 2 h at 4 ℃, the Eppendorf tube was centrifuged at 13,000*g* for 10 min at 4 ℃. The supernatant was transferred to a new Eppendorf tube, and the pellet was re-extracted with 500 μL of 100% MeOH. The secondary extract supernatant was combined with 80% MeOH extract supernatant, and was dried under nitrogen gas. Then 500-μL sodium acetate (0.1 M, pH 5.5) was used to dissolve the pellet. 250-μL suspension was used to determine the contents of free SA. The remaining 250 μL suspension was added with 10 μL of β-glucosidase (0.2 U μL^−1^), and hydrolyzed in the water bath at a 37 °C for 2 h. The sample was treated in boiling water for 5 min, and centrifuged at 13,000*g* for 10 min at 4 °C to remove the denatured enzyme. The supernatant was used for quantification of total SA, 2,3-DHBA, and 2,5-DHBA.

According to the previously described method [23], an Agilent 1260 HPLC system (Agilent Technologies, USA) coupled with a DAD detector and a fluorescence detector and a Zorbax SB-C18 column (4.6 × 250 mm, 5 μm; Agilent Technologies, USA) was used for the metabolite analysis. The mobile phases were composed of sodium acetate (0.2 M, pH 5.5) and MeOH. The gradient conditions of mobile phase were as follows: methanol gradient maintained at 3% for 12 min, linearly increased to 7% at 12.5 min, and maintained to 38 min, then decreased to 3% after 1 min. After the system was equilibrated for 7 min, the next injection was carried out. SA was detected by the fluorescence detector with 296-nm excitation wavelength and 410-nm emission wavelength, and 2,5-DHBA was detected by the fluorescence detector with 320-nm excitation wavelength and 449-nm emission wavelength. 2,3-DHBA was detected with a DAD detector at 223 nm. According to the standard curve and sample peak area, the concentration was calculated.

### Chlorophyll and SA detoxification assays

Chlorophyll assay was performed based on a previously described method [22]. Chlorophyll was extracted by acetone and determined by a spectrophotometer. Firstly, fresh leaves weighted as W were cut into strips and put into 3.5 mL 80% acetone (V/V). These samples were treated for 24 h under dark conditions at room temperature to completely dissolve chlorophyll in acetone. Then Nanodrop (Thermo fisher, USA) was used to determine the absorbance values at 645 nm (chlorophyll *b* absorption peak) and 663 nm (chlorophyll *a* absorption peak), which were denoted as A645 and A663. Finally, the concentration of chlorophyll in each solution was calculated by formula, C (mg/L) = (20.2 × A645 + 8.02 × A663); and the total chlorophyll content in leaves per unit fresh weight was calculated by formula, M (mg/g) = (C × 3.5)/(1000 × W).

To test the response of *Arabidopsis* plants to SA toxicity, the seeds of WT, *NahG*, and *S5Hpro::EGFP-S3H* transgenic plants were grown on 1/2 MS medium for 4 days and then transplanted to 1/2MS medium with 100 μM sodium salicylate for additional 7 days. The photos were taken and the chlorophyll content was quantified following a method described above.

### Fv/Fm assay and rosette leaf diameter measurements

The plants in the same growth state were selected and treated in dark environment for 30 min. Then the Fv/Fm value of the 5–6th rosette leaves were measured by Chlorophyll Fluorometer OS1P (Opti-Sciences, USA). The same position of each leaf was selected for measurement. Four biological replicates were determined for each line. The well-growing plants were selected for photographing, and the largest diameter of *Arabidopsis* rosette leaves were measured by using the software of Image J.

### RNA extraction and gene expression analyses

Total RNA was extracted using a TaKaRa minibest universal RNA extraction kit, and cDNAs were synthesized using HiScript Q RT supermix for quantitative qPCR (+ gDNA wiper; R123-01; Vazyme). qPCR was performed using Accupower Super qPCR kit (APS01; SibEnzyme) on an ABIPRISM7700 system (Applied Biosystems, USA). *ACTIN2* was used as an internal control in the analysis of the qRT-PCR data from whole leaves. The qPCR primers included *S3H*-qPCR-F (5′-TTCATCGTCAATATCGGCGAC-3′) and *S3H*-qPCR-R (5′-ATCGATAACCGCTCGTTCTCG-3′) for *S3H*, *PR1*-qPCR-F (5′-CGAAAGCTCAAGATAGCCCACA-3′) and *PR1*-qPCR-R (5′-TTCTGCGTAGCTCCGAGCATAG-3′) for *PR1*, *PR2*-qPCR-F (5′-GCTTCCTTCTTCAACCACACAGC-3′) and *PR2*-qPCR-R (5′-CGTTGATGTACCGGAATCTGAC-3′) for *PR2*, *ACTIN2*-qPCR-F (5′-GGTAACATTGTGCTCAGTGGTGG-3′) and *ACTIN2*-qPCR-R (5′-CTCGGCCTTGGAGATCCACATC-3′) for *ACTIN2*.

### Accession numbers

Sequence data for most genes studied in this article can be found in the *Arabidopsis* Genome Initiative database under the following accession numbers: *S3H* (AT4G10500); *DMR6 *(*S5H*) (AT5G24530); *ACTIN2* (AT3G18780); *PR1* (AT2G14610); *PR2* (AT3G57260).

## Supplementary Information

**Additional file 1: Figure S1.** Overexpression of SA 3-hydroxylase under the control of CaMV 35S promoter. **a** Vector map for *S3H* gene overexpression under the CaMV 35S promoter. The map was prepared by SnapGene. **b** The principle of SA catabolism to 2,3-DHBA in *35Spro::EGFP-S3H* transgenic plants. The 35S promoter constitutively drives the expression of *EGFP-S3H*. The enzyme S3H can convert SA into 2,3-DHBA and reduce the SA levels. **Figure S2.** Quantification of SA in the *35Spro::EGFP-S3H* and *S5Hpro::EGFP-S3H* transgenic plants. **a** Morphological phenotypes of WT, *NahG*, and representative *35Spro::EGFP-S3H* transgenic plants at 28 DAG. **b** Morphological phenotypes of WT, *NahG*, and representative *S5Hpro::EGFP-S3H* transgenic plants at 28 DAG. **c**, **d** Relative levels of free SA (**c**) and total SA (**d**) in WT, *NahG*, and representative *35Spro::EGFP-S3H* transgenic plants. **e**, **f** Relative content of free SA (**e**) and total SA (**f**) in WT, *NahG*, and representative *S5Hpro::EGFP-S3H* transgenic plants. The data are means ± SE (n = 3 biological replicates); FW, fresh weight. Scale bar = 2 cm. Statistical differences among replicates are labeled with different letters (*P* < 0.05, one-way ANOVA and post-hoc Tukey’s test). **Figure S3.** Expression of *S3H* in the single-copy *S5Hpro::EGFP-S3H* transgenic plants. Quantification of the *S3H* expression in WT, *NahG*, and *S5Hpro::EGFP-S3H* transgenic plants at 21 DAG by qRT-PCR. The data are means ± SE (n = 3 biological replications). Statistical differences among replicates are labeled with different letters (*P* < 0.05, one-way ANOVA and post-hoc Tukey’s test). **Figure S4.** Growth and morphological phenotypes in *S5Hpro::EGFP-S3H* transgenic plants of Ws and L*er* accessions. **a** Morphological phenotype of *S5Hpro::EGFP-S3H* transgenic plants of Ws accession at 28 DAG, Bar = 2 cm. **b** Morphological phenotypes of *S5Hpro::EGFP-S3H* transgenic plants of L*er* accession at 28 DAG, Bar = 2 cm. **c** Quantification of the rosette leaf diameters from plants in (**a**). **d** Quantification of the rosette leaf diameters from plants in (**b**). The data are presented as means ± SE (n ≥ 5 biological replicates). Statistical differences among replicates are labeled with different letters (*P* < 0.05, one-way ANOVA and post-hoc Tukey’s test). **Figure S5.** Spatial, temporal, and inducible expression patterns of *S5H/DMR6* gene in *Arabidopsis*. **a**–**d** Expression patterns of *S5H/DMR6* (AT5G24530) in different tissues (**a**), different developmental stages (**b**), under different abiotic stress (**c**), and biotic stress (**d**). **e** Expression patterns of *S5H* induced by various abiotic and biotic stress treatments for certain time. The data were extracted from RNAseq database (http://ipf.sustech.edu.cn/pub/athrna/).

## Data Availability

The materials in this study are available from the corresponding author on reasonable request.
