# Supplementary material for: Generation of the salicylic acid deficient Arabidopsis via a synthetic salicylic acid hydroxylase expression cassette
Source: Plant Methods. 2022 Jun 28;18:89. doi: 10.1186/s13007-022-00922-x (PMC9238041; doi:10.1186/s13007-022-00922-x)
Supplement: Supplementary file 1 — Additional file 1: Figure S1. Overexpression of SA 3-hydroxylase under the control of CaMV 35S promoter. a Vector map for S3H gene overexpression under the CaMV 35S promoter. The map was prepared by SnapGene. b The principle of SA catabolism to 2,3-DHBA in 35Spro::EGFP-S3H transgenic plants. The 35S promoter constitutively drives the expression of EGFP-S3H. The enzyme S3H can convert SA into 2,3-DHBA and reduce the SA levels. Figure S2. Quantification of SA in the 35Spro::EGFP-S3H and S5Hpro::EGFP-S3H transgenic plants. a Morphological phenotypes of WT, NahG, and representative 35Spro::EGFP-S3H transgenic plants at 28 DAG. b Morphological phenotypes of WT, NahG, and representative S5Hpro::EGFP-S3H transgenic plants at 28 DAG. c, d Relative levels of free SA (c) and total SA (d) in WT, NahG, and representative 35Spro::EGFP-S3H transgenic plants. e, f Relative content of free SA (e) and total SA (f) in WT, NahG, and representative S5Hpro::EGFP-S3H transgenic plants. The data are means ± SE (n = 3 biological replicates); FW, fresh weight. Scale bar = 2 cm. Statistical differences among replicates are labeled with different letters (P < 0.05, one-way ANOVA and post-hoc Tukey’s test). Figure S3. Expression of S3H in the single-copy S5Hpro::EGFP-S3H transgenic plants. Quantification of the S3H expression in WT, NahG, and S5Hpro::EGFP-S3H transgenic plants at 21 DAG by qRT-PCR. The data are means ± SE (n = 3 biological replications). Statistical differences among replicates are labeled with different letters (P < 0.05, one-way ANOVA and post-hoc Tukey’s test). Figure S4. Growth and morphological phenotypes in S5Hpro::EGFP-S3H transgenic plants of Ws and Ler accessions. a Morphological phenotype of S5Hpro::EGFP-S3H transgenic plants of Ws accession at 28 DAG, Bar = 2 cm. b Morphological phenotypes of S5Hpro::EGFP-S3H transgenic plants of Ler accession at 28 DAG, Bar = 2 cm. c Quantification of the rosette leaf diameters from plants in (a). d Quantification of the rosette le [file 13007_2022_922_MOESM1_ESM.pdf]

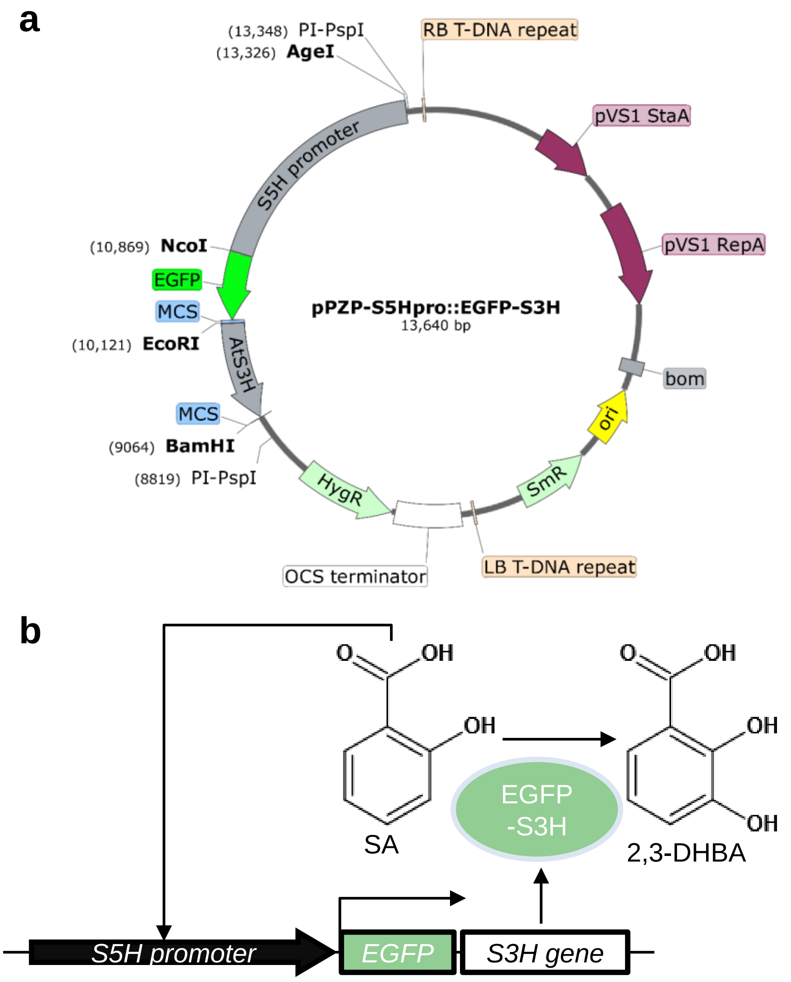

Fig. 1 Design of a synthetic SA hydroxylase expression cassette catalyzing SA to 2,3-DHBA. **a,** Vector map for *S3H* gene overexpression. The *S5H* promoter of *Arabidopsis* was cloned and constructed into the plant expression vector (pPZP-RCS2) to drive the expression of *EGFP-S3H*. The map was prepared by SnapGene. **b,** The principle of a feedback loop for SA catabolism to 2,3-DHBA in *S5Hpro::EGFP-S3H*transgenic plants. The *S5H* promoter can be induced by SA and then drive the expression of *EGFP-S3H*. The expressed S3H enzyme can convert SA into 2,3-DHBA and reduce the SA levels.

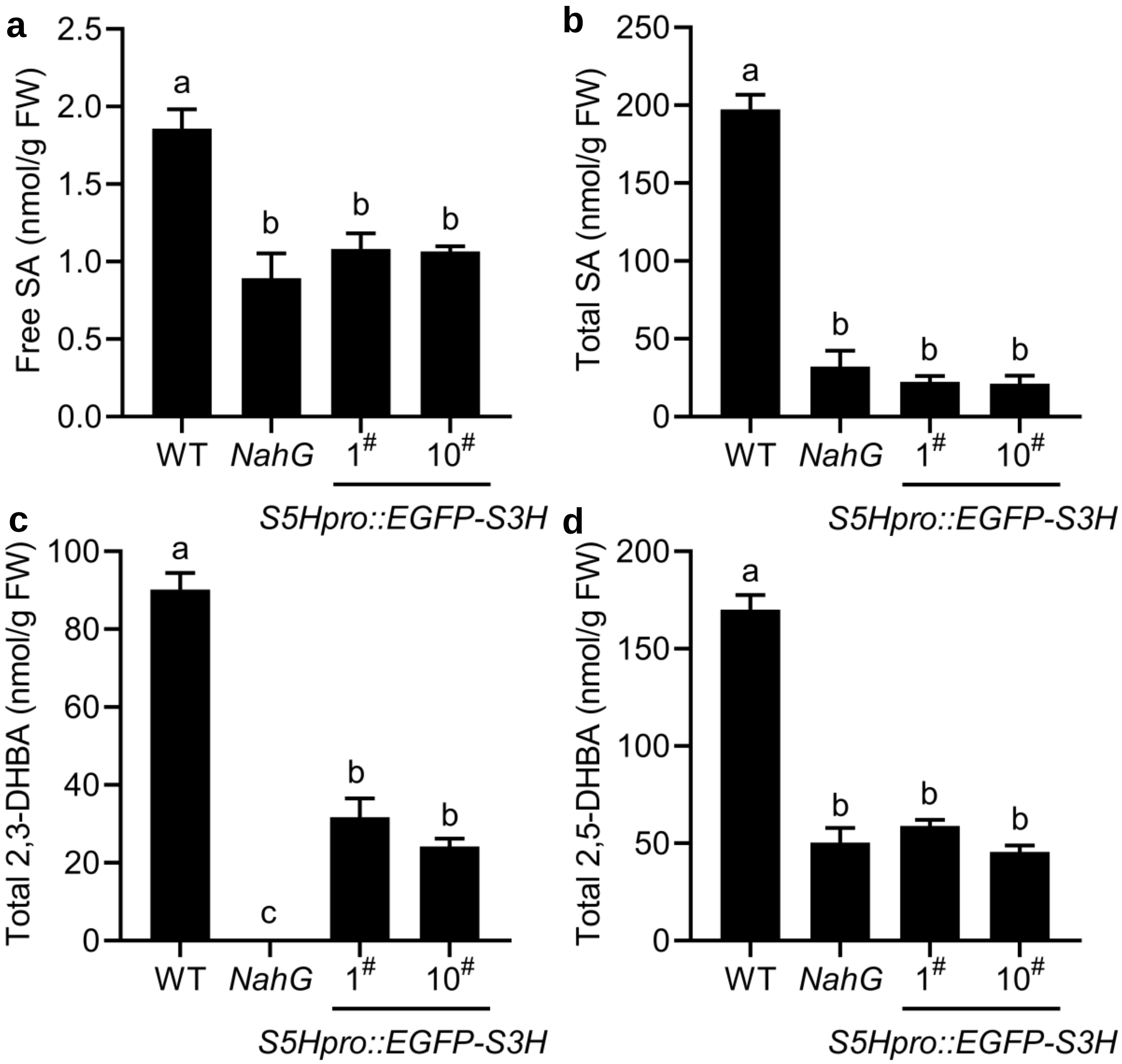

Fig. 2 Quantification of SA, 2,3-DHBA, and 2,5-DHBA in the single-copy *S5Hpro::EGFP-S3H* transgenic plants.

a–**d** the levels of free SA (**a**), total SA (**b**), total 2,3-DHBA (**c**), and total 2,5-DHBA (**d**) in WT, *NahG* and single-copy *S5Hpro::EGFP-S3H* transgenic plants at 28 DAG. The data are means ± SE (n = 3 biological replications); FW, fresh weight. Statistical differences among replicates are labeled with different letters ($P < 0.05$, one-way ANOVA and post-hoc Tukey's test).

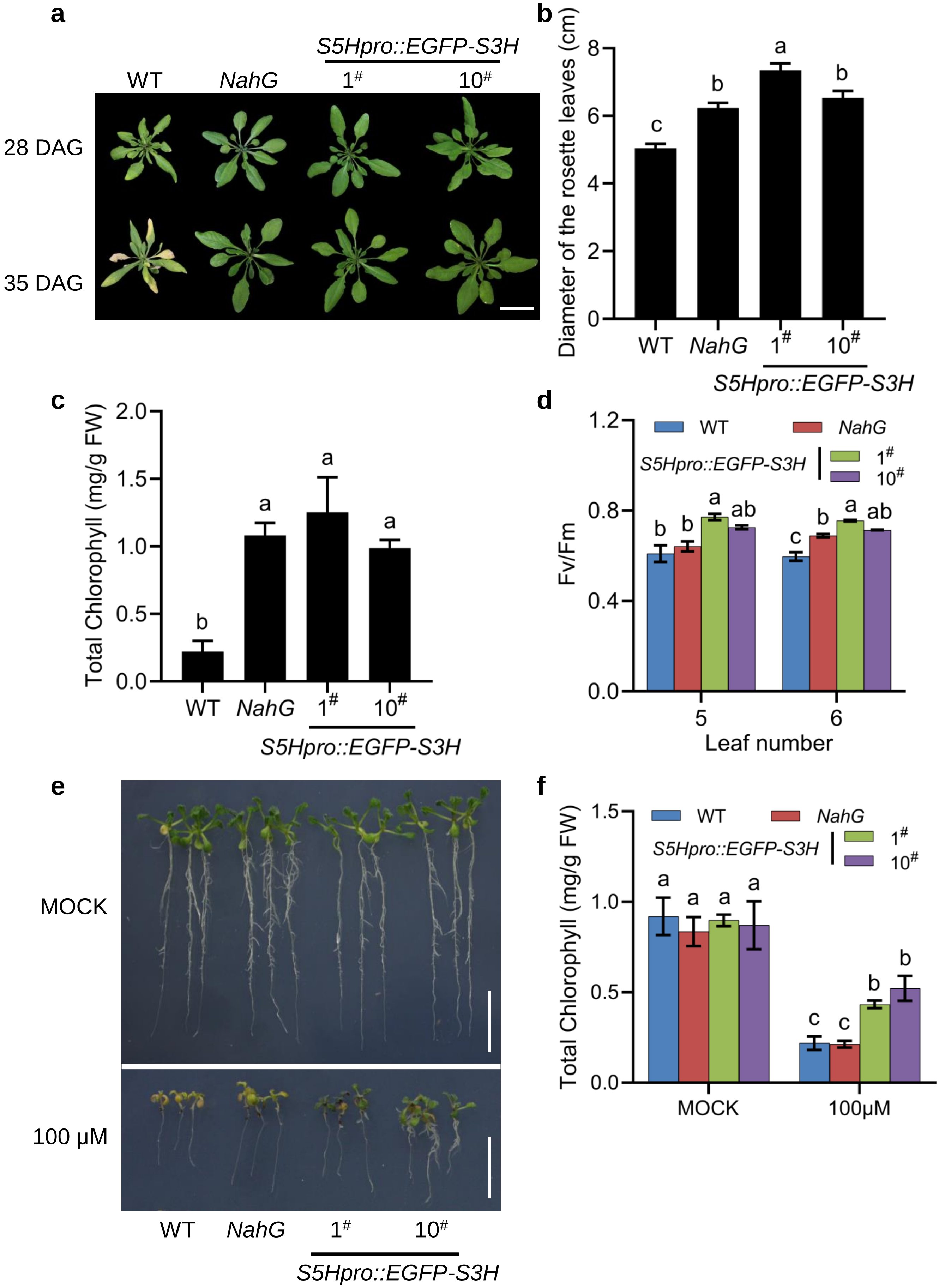

a
S5Hpro::EGFP-S3H
WT
NahG
1#
10#
28 DAG
35 DAG
b
Diameter of the rosette leaves (cm)
0
2
4
6
8
c
b
a
b
WT
NahG
1#
10#
S5Hpro::EGFP-S3H
c
Total Chlorophyll (mg/g FW)
0.0
0.5
1.0
1.5
2.0
b
a
a
a
WT
NahG
1#
10#
S5Hpro::EGFP-S3H
d
WT
NahG
S5Hpro::EGFP-S3H
1#
10#
Fv/Fm
0.0
0.4
0.8
1.2
b
b
a
ab
c
b
a
ab
5
6
Leaf number
e
MOCK
100 μM
WT
NahG
1#
10#
S5Hpro::EGFP-S3H
f
WT
NahG
S5Hpro::EGFP-S3H
1#
10#
Total Chlorophyll (mg/g FW)
0.0
0.5
1.0
1.5
a
a
a
a
c
c
b
b
MOCK
100μM

Fig. 3 Growth and developmental phenotypes of the *S5Hpro::EGFP-S3H* transgenic plants.

a Morphological phenotypes of WT, *NahG* and *S5Hpro::EGFP-S3H* transgenic plants at 28 and 35 DAG, Bar = 2 cm. **b** Quantification of the rosette leaf diameters from plant of (**a**) at 28 DAG. The data are presented as means ± SE (n ≥ 10 biological replications). **c** Quantification of chlorophyll content in the 5th~6th leaves from plant of (**a**) at 35 DAG, the data are means ± SE (n = 4 biological replications); FW, fresh weight. **d** Fv/Fm of the 5th~6th leaves from plant (**a**) at 35 DAG, the data are means ± SE (n = 4 biological replications). **e** Phenotypes of WT, *NahG* and *S5Hpro::EGFP-S3H* transgenic plants grown on 1/2MS medium with or without 100 μM sodium salicylate. Bar = 1 cm. **f** Quantification of chlorophyll content from plant of (**e**), the data are means ± SE (n = 4 biological replications); FW, fresh weight. Statistical differences among replicates are labeled with different letters (*P* < 0.05, one-way ANOVA and post-hoc Tukey's test).

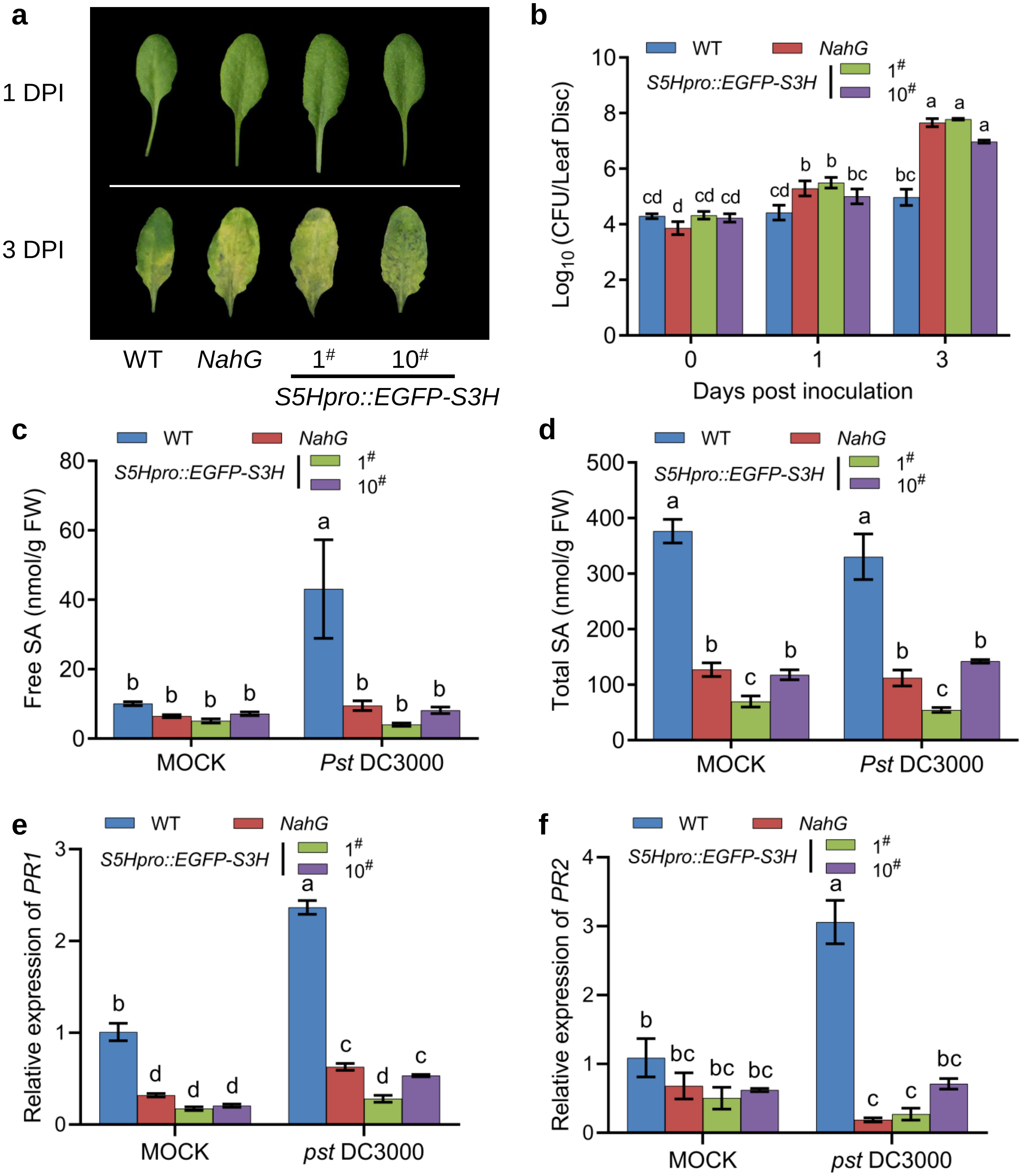

Fig. 4 Pathogen resistance of the *S5Hpro::EGFP-S3H* transgenic plants to *Pst* DC3000.
a The disease symptoms of WT, *NahG*, single-copy *S5Hpro::EGFP-S3H* transgenic plants 3 days after *Pst* DC3000 infection. **b** Quantification of the growth of *Pst* DC3000 in plants of (**a**) at 0, 1, 3 days post inoculation (DPI). The data are means ± SE (n = 6 biological replications). **c, d** The levels of free SA (**c**) and total SA (**d**) in WT, *NahG* and *S5Hpro::EGFP-S3H* transgenic plants after *Pst DC3000* infection. The data are means ± SE (n = 4 biological replications). **e, f** Quantification of the *PR1* (**e**) and *PR2* (**f**) expression in WT, *NahG* and *S5Hpro::EGFP-S3H* transgenic plants after *Pst* DC3000 infection by qPCR. The data are means ± SE (n = 3 biological replications). Statistical differences among replicates are labeled with different letters ($P < 0.05$, one-way ANOVA and post-hoc Tukey's test).

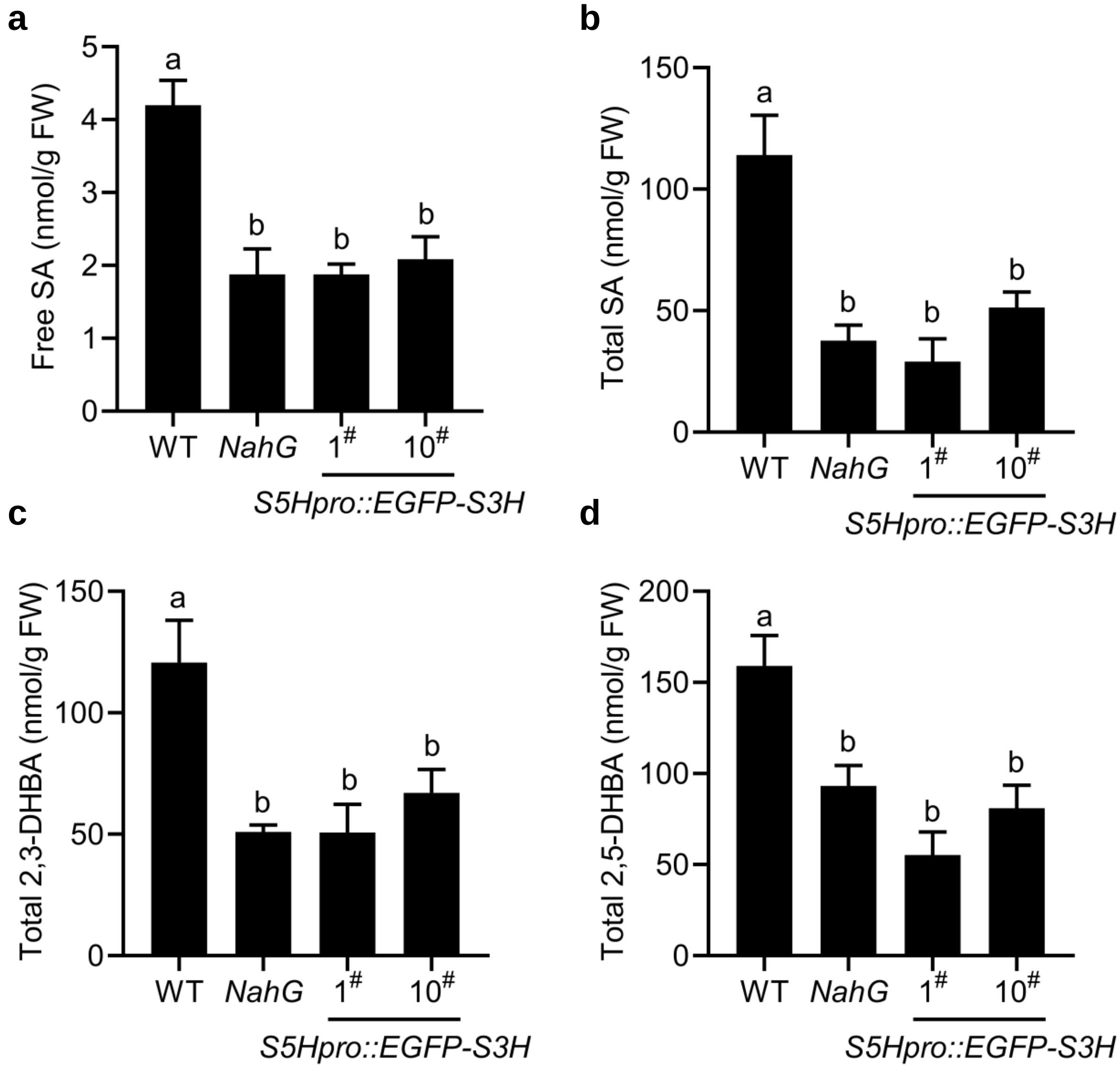

Fig. 5 Quantification of SA, 2,3-DHBA and 2,5-DHBA in *S5Hpro::EGFP-S3H* transgenic plants at T5 generation.

a–d Quantification of free SA (**a**), total SA (**b**), total 2,3-DHBA (**c**) and total 2,5-DHBA (**d**) from WT, *NahG*, and single-copy *S5Hpro::EGFP-S3H* transgenic lines at T5 generation at 35 DAG. The data are means ± SE (n=4 biological replications); FW, fresh weight. Statistical differences among replicates are labeled with different letters ($P < 0.05$, one-way ANOVA and post-hoc Tukey's test).

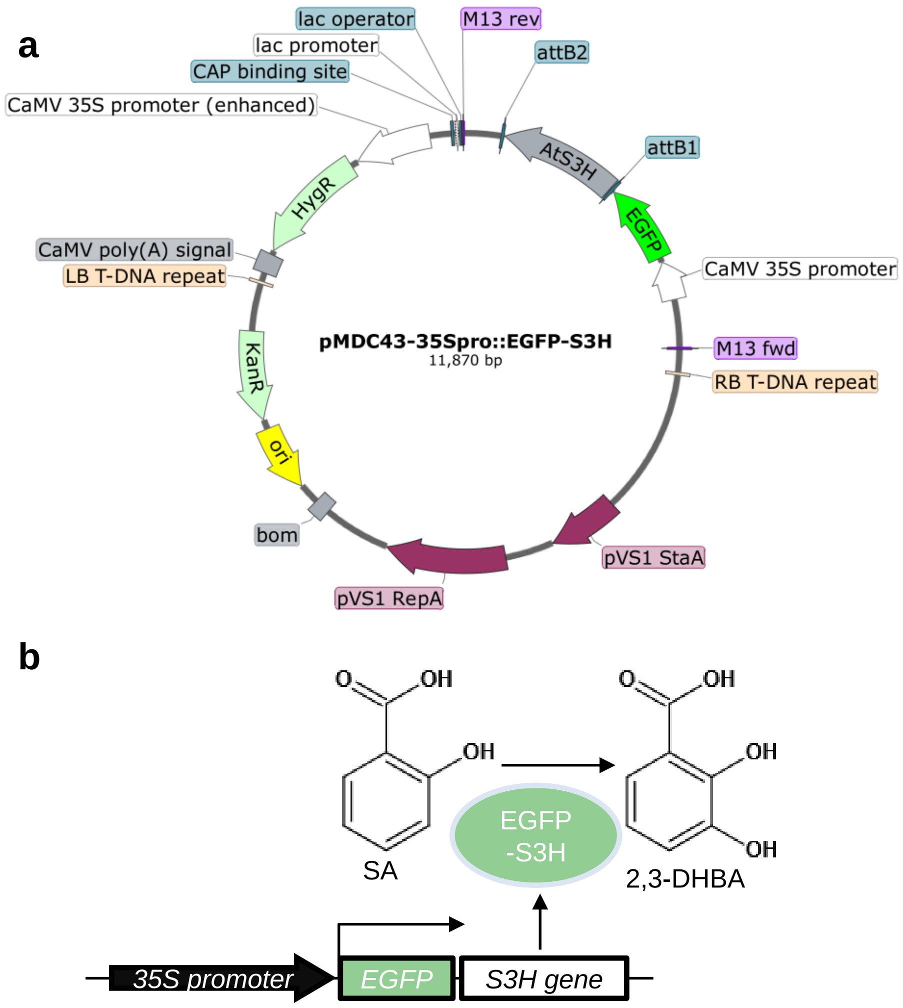

Fig. S1 Overexpression of SA 3-hydroxylase under the control of CaMV 35S promoter.
a Vector map for *S3H* gene overexpression under the CaMV 35S promoter. The map was prepared by SnapGene. **b** The principle of SA catabolism to 2,3-DHBA in *35Spro::EGFP-S3H* transgenic plants. The 35S promoter constitutively drives the expression of *EGFP-S3H*. The enzyme S3H can convert SA into 2,3-DHBA and reduce the SA levels.

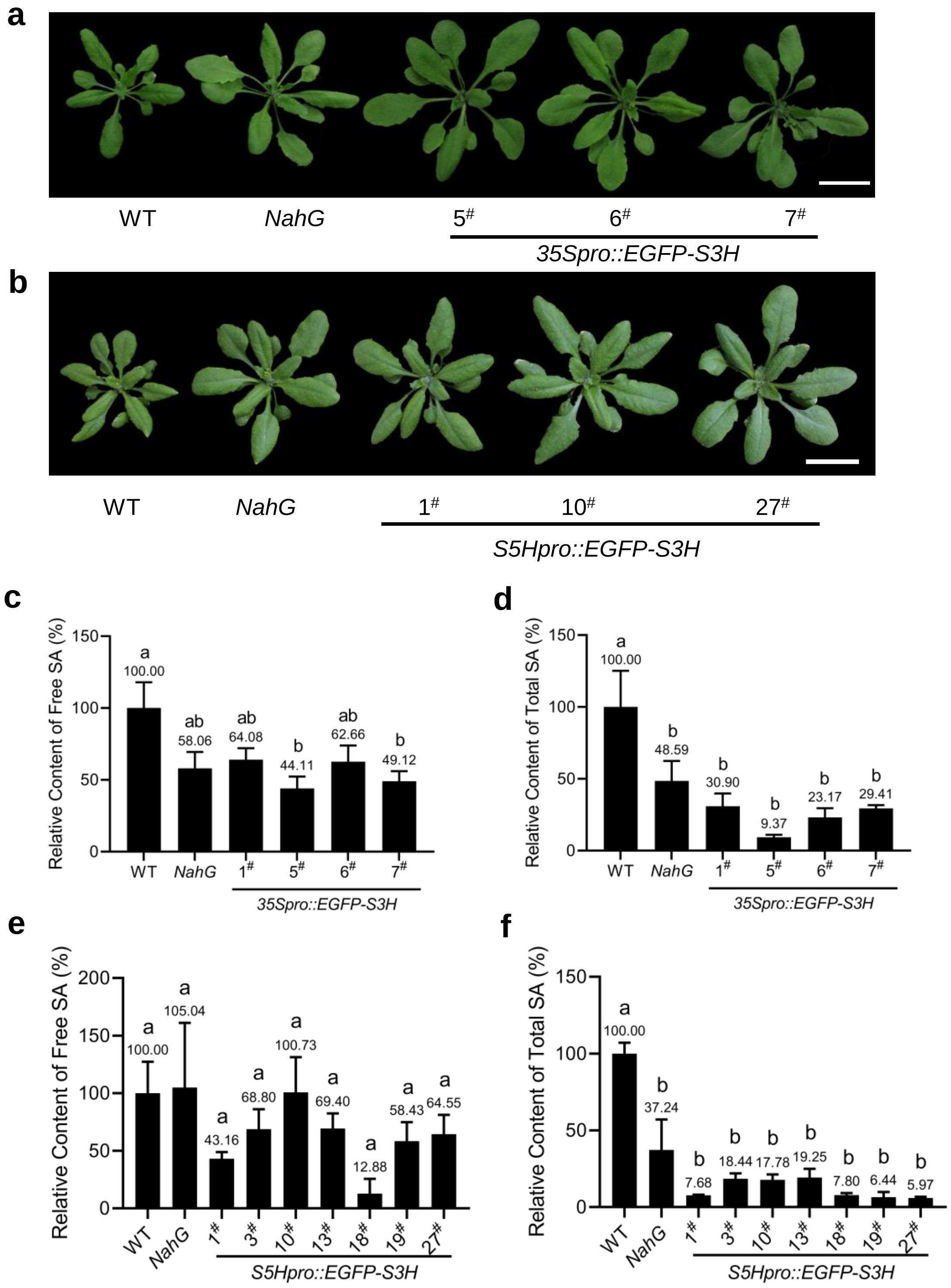

Fig. S2 Quantification of SA in the *35Spro::EGFP-S3H* and *S5Hpro::EGFP-S3H* transgenic plants.

a Morphological phenotypes of WT, *NahG*, and representative *35Spro::EGFP-S3H* transgenic plants at 28 DAG. **b** Morphological phenotypes of WT, *NahG*, and representative *S5Hpro::EGFP-S3H* transgenic plants at 28 DAG. **c, d** Relative levels of free SA (**c**) and total SA (**d**) in WT, *NahG*, and representative *35Spro::EGFP-S3H* transgenic plants. **e, f** Relative content of free SA (**e**) and total SA (**f**) in WT, *NahG*, and representative *S5Hpro::EGFP-S3H* transgenic plants. The data are means ± SE (n = 3 biological replications); FW, fresh weight. Scale bar = 2 cm. Statistical differences among replicates are labeled with different letters ($P < 0.05$, one-way ANOVA and post-hoc Tukey's test).

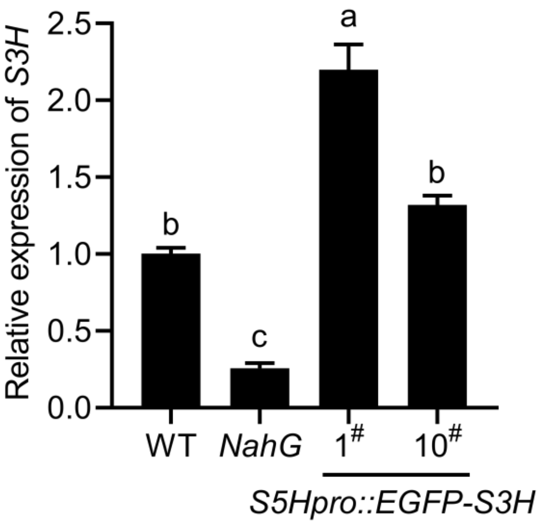

Fig. S3 Expression of *S3H* in the single-copy *S5Hpro::EGFP-S3H* transgenic plants .
Quantification of the *S3H* expression in WT, *NahG* and *S5Hpro::EGFP-S3H* transgenic plants at 21 DAG by qRT-PCR. The data are means ± SE (n = 3 biological replications). Statistical differences among replicates are labeled with different letters ($P < 0.05$, one-way ANOVA and post-hoc Tukey's test).

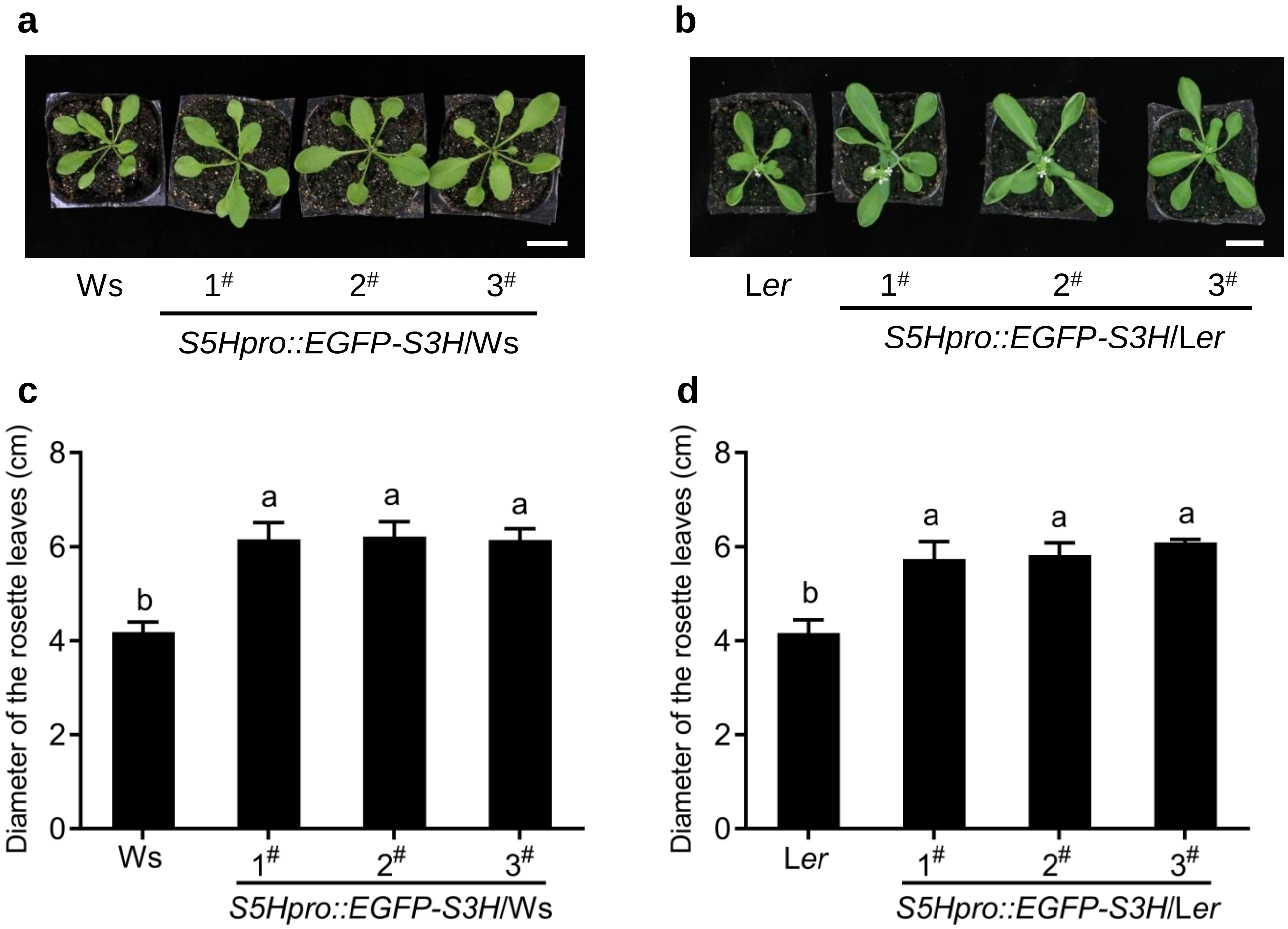

Fig. S4 Growth and morphological phenotypes in *S5Hpro::EGFP-S3H* transgenic plants of Ws and L*er* accessions.

a Morphological phenotype of *S5Hpro::EGFP-S3H* transgenic plants of Ws accession at 28 DAG, Bar = 2 cm. **b** Morphological phenotypes of *S5Hpro::EGFP-S3H* transgenic plants of L*er* accession at 28 DAG, Bar = 2 cm. **c** Quantification of the rosette leaf diameters from plants in (**a**). **d** Quantification of the rosette leaf diameters from plants in (**b**). The data are presented as means ± SE (n ≥ 5 biological replications). Statistical differences among replicates are labeled with different letters ($P < 0.05$, one-way ANOVA and post-hoc Tukey’s test).

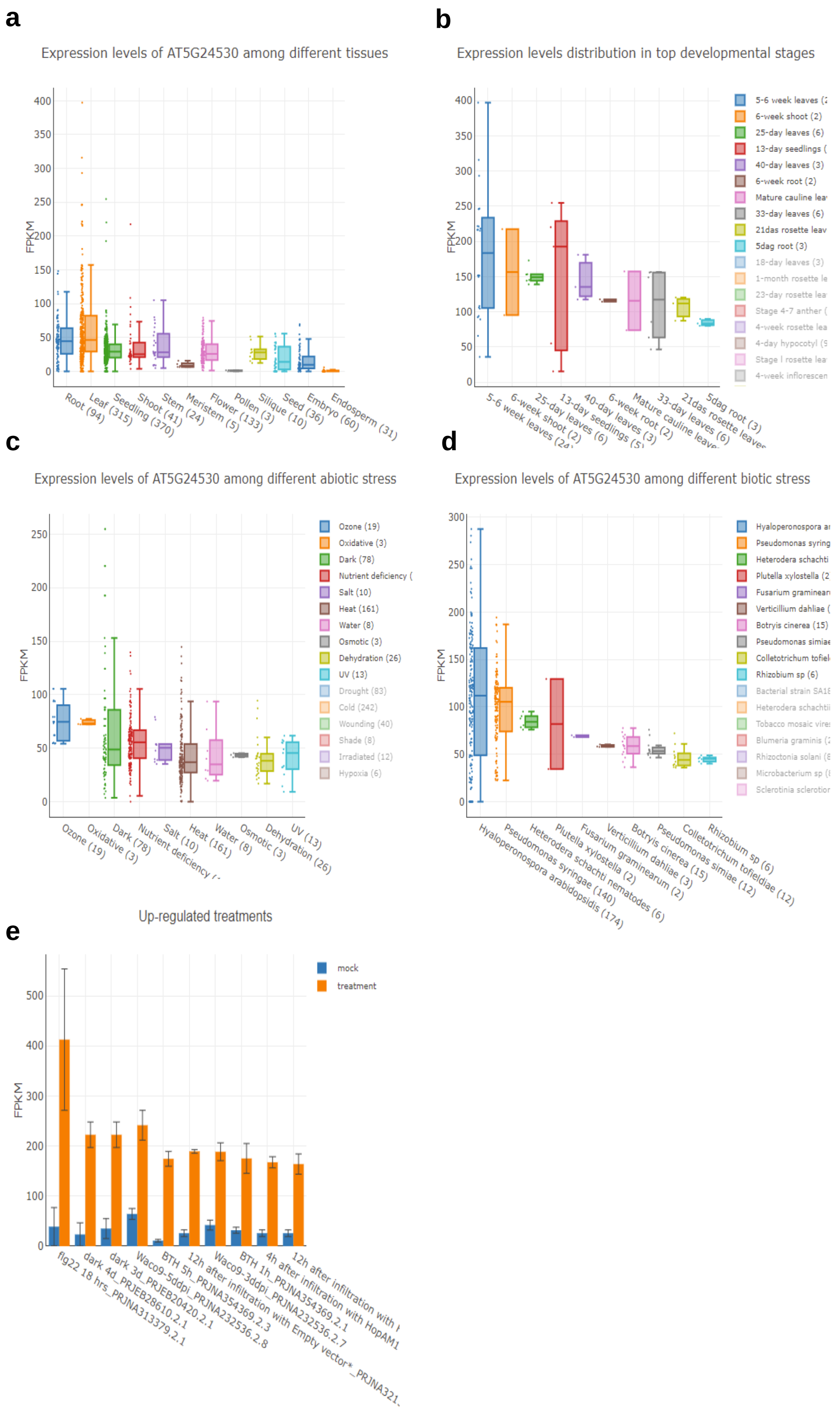

Fig. S5 Spatial, temporal, and inducible expression patterns of *S5H/DMR6* in *Arabidopsis*.

a–d Expression patterns of *S5H/DMR6* (AT5G24530) in different tissues (**a**), different developmental stages (**b**), and under different abiotic stress (**c**) and biotic stress (**d**). **e** Expression patterns of *S5H* induced by various abiotic and biotic stress treatments for certain time. The data were extracted from RNAseq database (http://ipf.sustech.edu.cn/pub/athrna/).
